# Supplementary material for: A presynaptic source drives differing levels of surround suppression in two mouse retinal ganglion cell types
Source: Nat Commun. 2024 Jan 18;15:599. doi: 10.1038/s41467-024-44851-w (PMC10796971; doi:10.1038/s41467-024-44851-w)
Supplement: Supplementary file 1 — Supplementary Information [file 41467_2024_44851_MOESM1_ESM.pdf]

A presynaptic source drives differing levels of surround suppression in two mouse retinal ganglion cell types.

David Swygart[1], Wan-Qing Yu[2], Shunsuke Takeuchi[3], Rachel R. O.L. Wong[2], Gregory W. Schwartz*[,1,4,5]

1. Northwestern University Interdepartmental Neuroscience Program
2. Department of Biological Structure, University of Washington
3. Department of Biological Sciences, Graduate School of Science, The University of Tokyo, Tokyo, Japan
4. Departments of Ophthalmology and Neuroscience, Feinberg School of Medicine, Northwestern University
5. Department of Neurobiology, Weinberg College of Arts and Sciences, Northwestern University
* Corresponding author: greg.schwartz@northwestern.edu

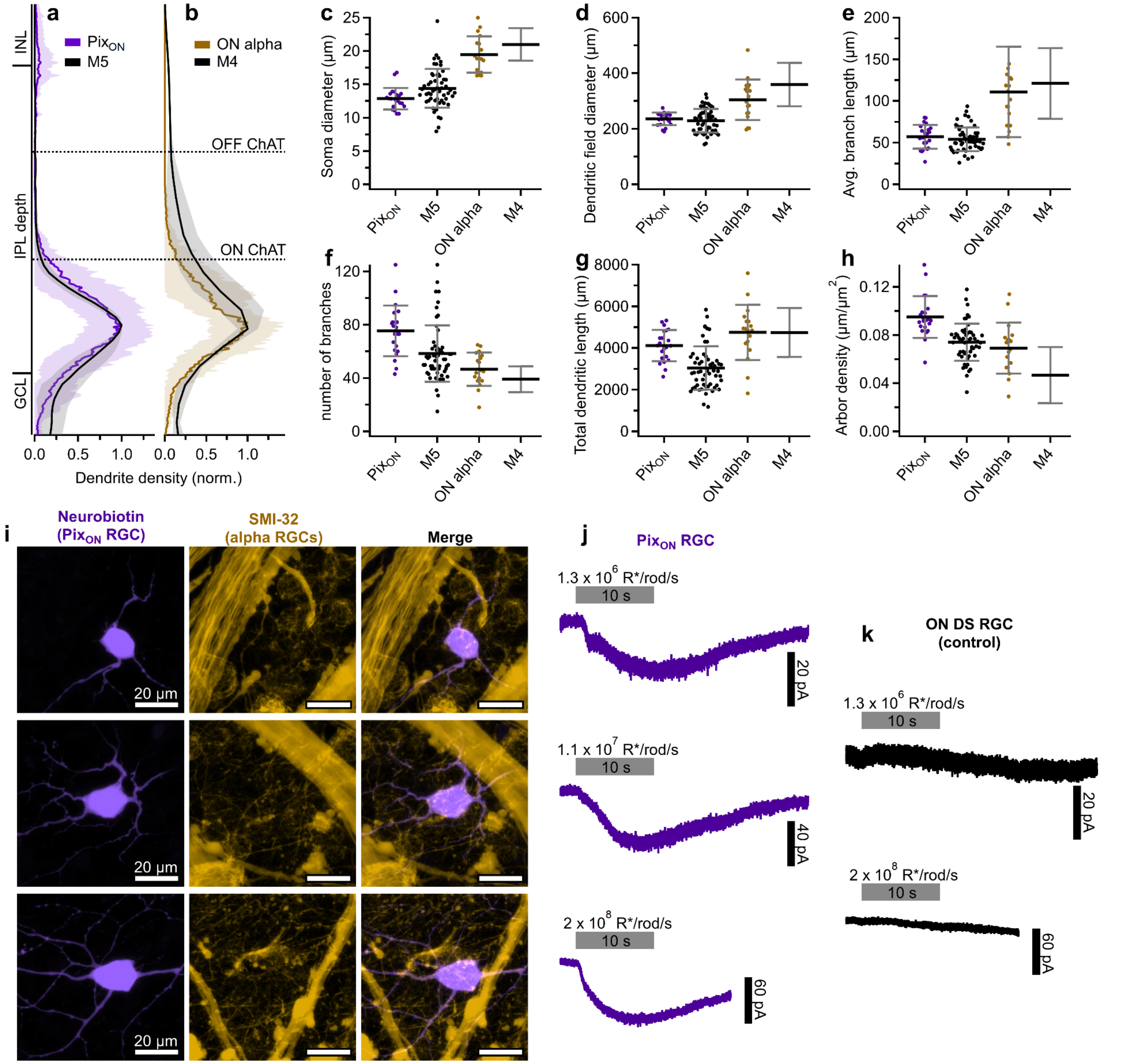

Supplementary Fig. 1 | Pix$_{ON}$ and ON alpha RGCs exhibit unique morphology and correspond to M5 and M4 RGCs.
a, Dendritic stratification of Pix$_{ON}$ (n=19) and M5 (n=2) RGCs within the inner nuclear layer (INL), inner plexiform layer (IPL), and ganglion cell layer (GCL). Dotted lines refer to the ON and OFF choline acetyltransferase (ChAT) bands used to determine stratification. Shading and error bars indicate ± standard deviation. **b**, Same as **a**, but comparing ON alpha (n=10) and M4 (n=2) dendritic stratification. **c**-**h**, Comparison of soma diameter (**c**), dendritic field diameter (**d**), average dendritic branch length (**e**), total number of dendritic branches (**f**), total dendritic length (**g**), and arbor density (**h**) between Pix$_{ON}$ (n=22), M5 (n=56), ON alpha (n=18), and M4 (n=27) RGC types. Arbor density (**h**) was calculated as the total dendritic length normalized by dendritic field area. Dots indicate data from individual cells. Bar plots indicate average ± std. **a-h**, M5 and M4 RGC morphological data were generously provided by Professor David Berson and are published in Stabio, et al. (2018) and Estevez et al. (2012), respectively. **i**, *En-face* view of three different Pix$_{ON}$ RGC somas visualized by neurobiotin fill (left), SMI-32 staining to mark alpha RGCs (middle), and merged images. **j**, Intrinsic photocurrents measured by voltage-clamp recordings (V_{CMD} = -60 mV) during pharmacological blockade of retinal synapses (L-AP4, DNQX, and D-AP5). Gray bars indicate a 10-second full-field light step. Light intensity is reported in rhodopsin isomerizations per rod per second (R*/rod/s). Currents were measured from the same cells as in **i**. **k**, Same as **j**, but recorded from ON direction-selective RGCs, which are not expected to exhibit intrinsic photocurrents. Source data are provided as a Source Data file.

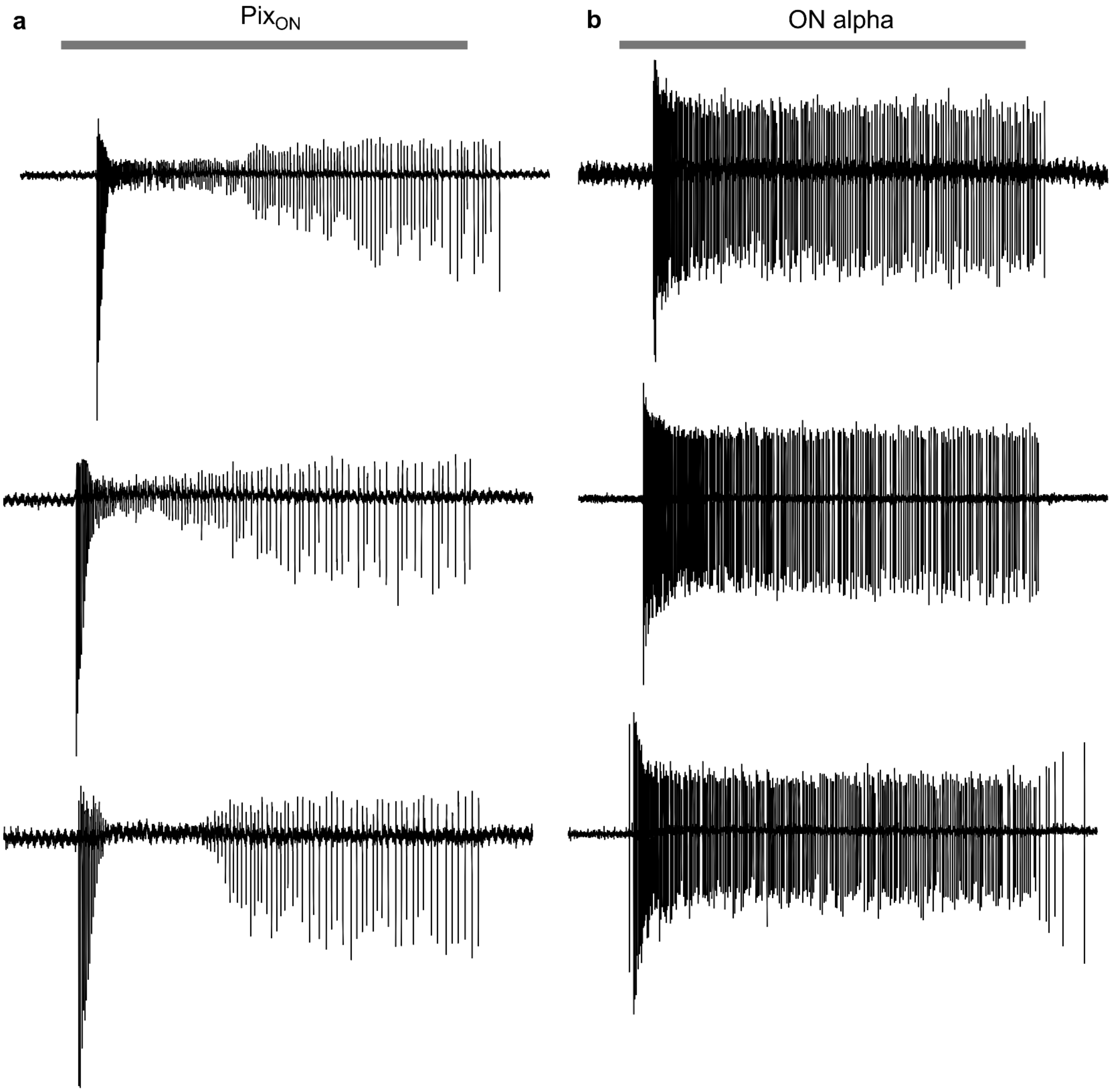

Supplementary Fig. 2 | Strong spike amplitude adaptation in Pix_{ON} RGC responses but not ON alpha RGC responses.
a, Cell attached recordings of three different Pix_{ON} RGCs in response to a preferred size spot stimulus. Gray bar indicates 1s stimulus presentation. **b**, Same as **a**, but spiking response recorded from three different ON alpha RGCs.

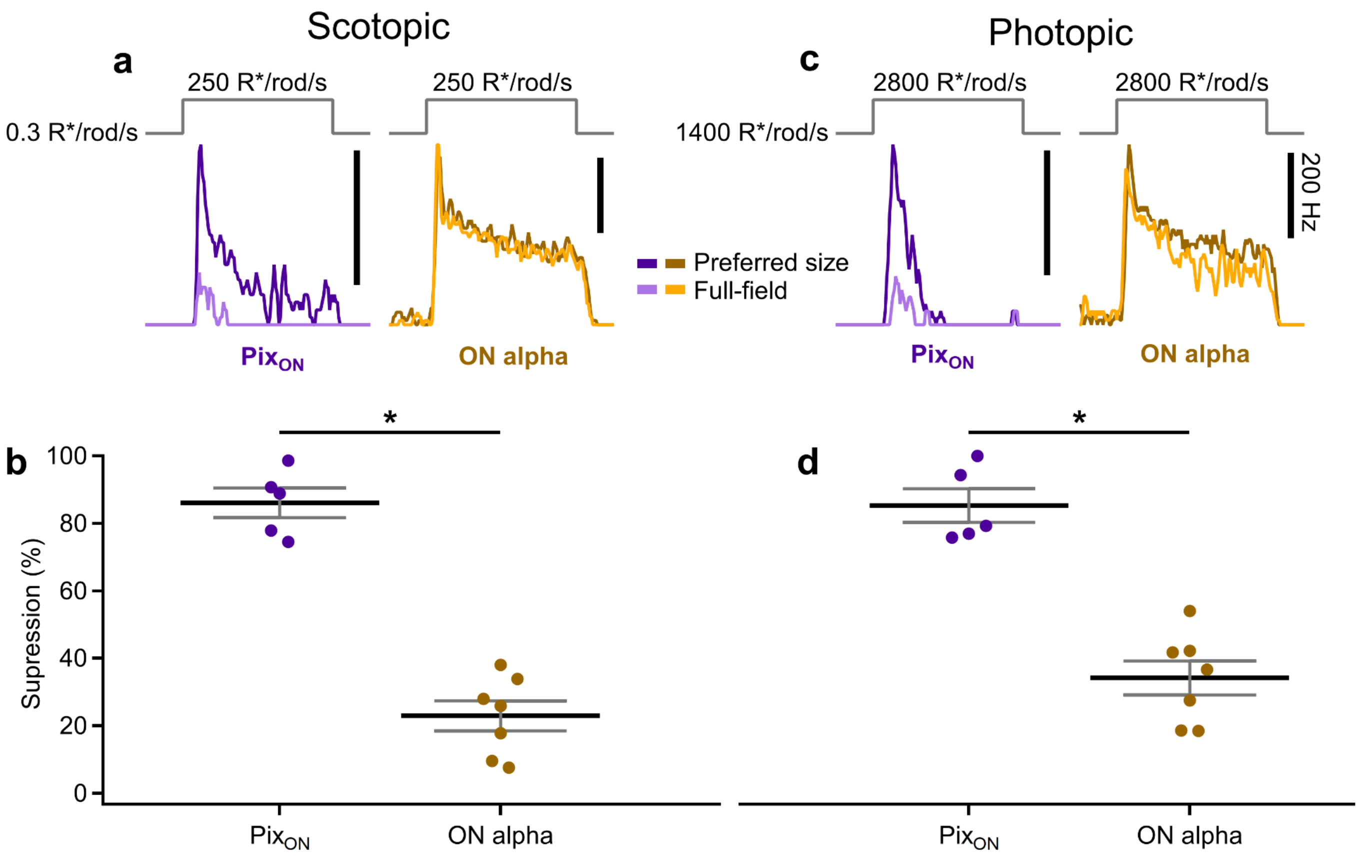

Supplementary Fig. 3 | Pix$_{ON}$ and ON alpha RGCs have differing levels of surround suppression in both scotopic and photopic conditions.

a, Example Pix$_{ON}$ (purple) and ON alpha (brown) peristimulus time histograms to preferred size and full-field light spot stimuli. The stimulus occurred within the scotopic luminance regime, stepping to a light intensity of 250 rhodopsin isomerizations per rod per second (R*/rod/s) from a background intensity of ~0.3 R*/rod/s for 1 second. **b**, Surround suppression in Pix$_{ON}$ (n=5) and ON alpha (n=7) RGCs to scotopic stimuli. Dots indicate data from individual cells. Bar plots indicate average ± s.e.m., *p<0.05, paired two-sample Student's *t*-test. **c**, Same as **a**, but the stimulus occurred within the photopic luminance regime, stepping from 1400 to 2800 R*/rod/s for 1 second. **d**, Surround suppression to photopic stimuli for the same cells as in **b**. Source data are provided as a Source Data file.

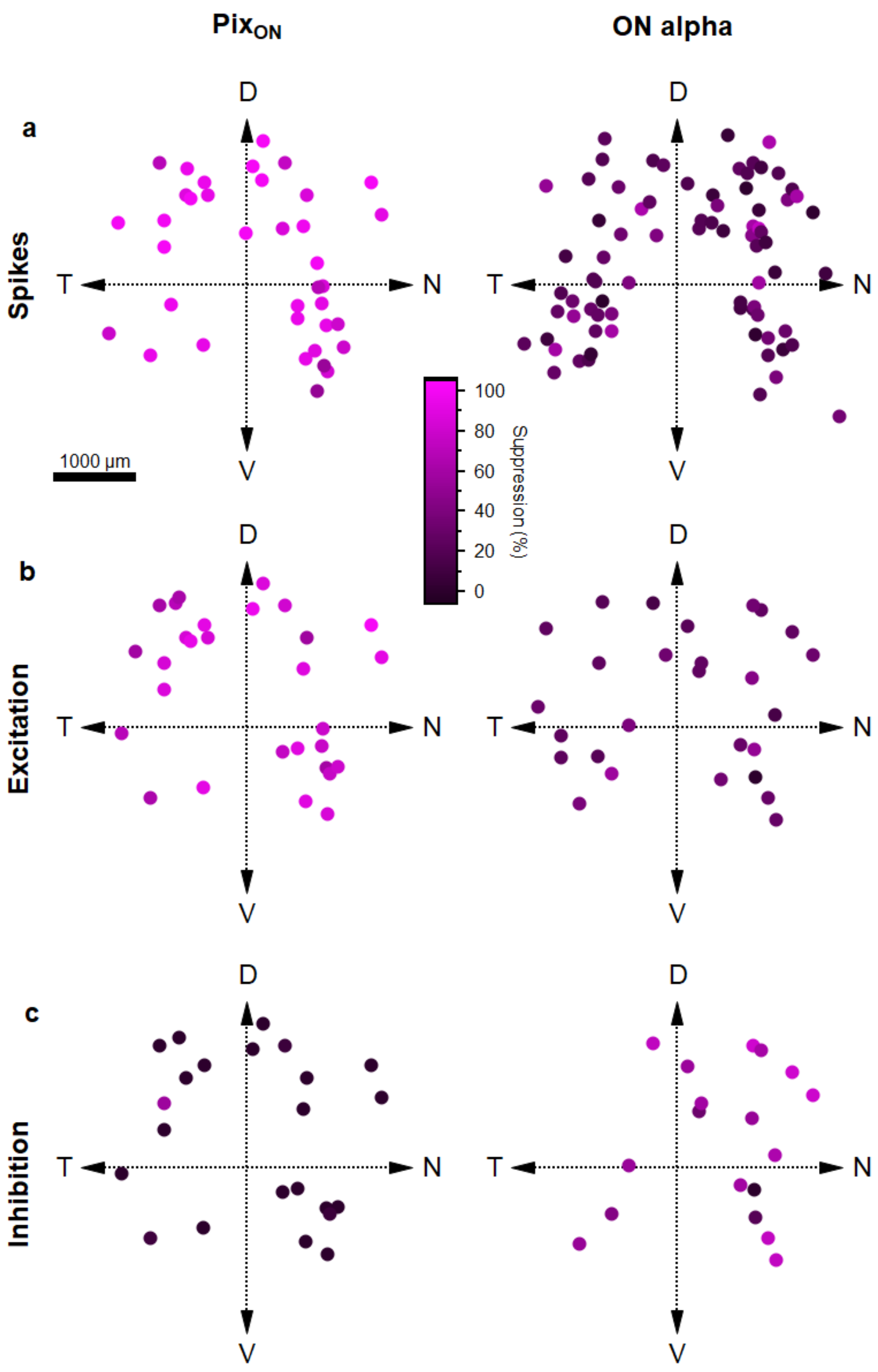

Supplementary Fig. 4 | Pix$_{ON}$ RGCs have stronger surround suppression than ON alpha RGCs across retinal locations.
a, Surround suppression of Pix$_{ON}$ (left) and ON alpha (right) spiking responses plotted by retinal location. Dots indicate the location of individual cells plotted on a dorsal (D) / ventral (V) / temporal (T) / nasal (N) coordinate scheme of the retina. Pix$_{ON}$ (n=38), ON alpha (n=79). **b**, Same as **a**, but for surround suppression of excitatory conductances. Pix$_{ON}$ (n=30), ON alpha (n=27). **c**, Same as **a**, but for surround suppression of inhibitory conductances. Pix$_{ON}$ (n=24), ON alpha (n=18). Source data are provided as a Source Data file.

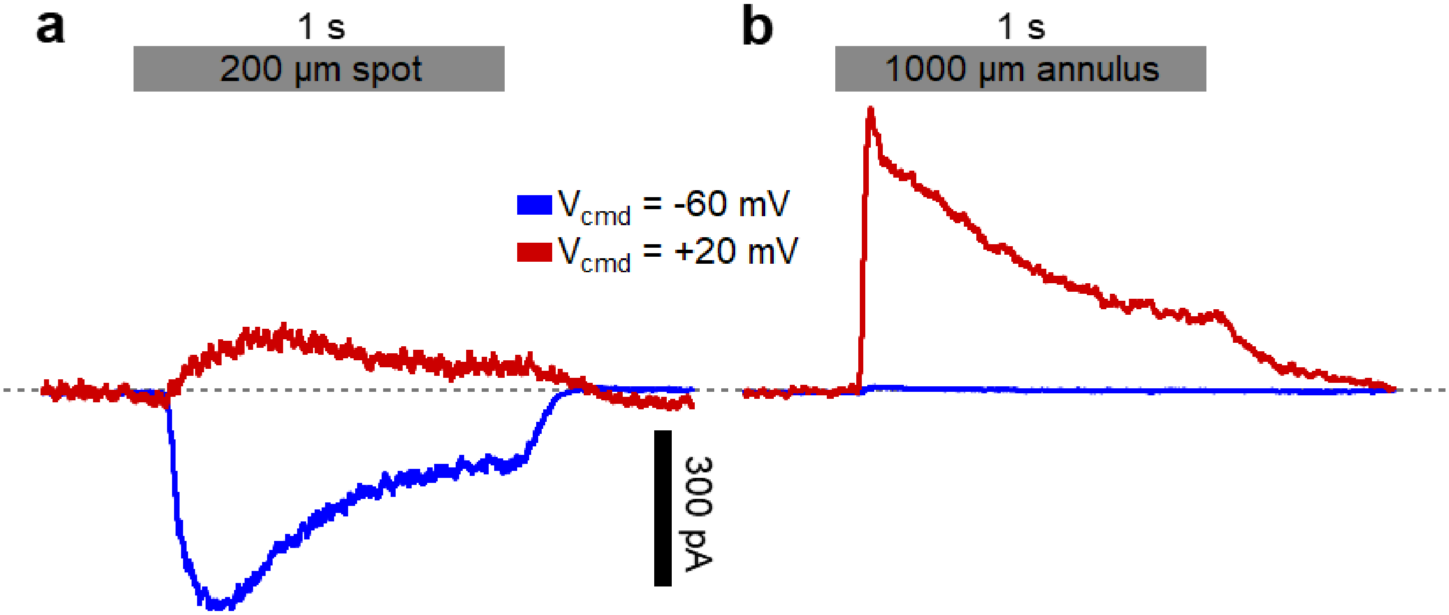

Supplementary Fig. 5 | Verification of voltage-clamp isolation of excitatory and inhibitory currents in a Pix$_{ON}$ RGC.
a, Synaptic currents evoked by a 1-second light step of a 200 µm diameter spot while voltage clamping at -60 mV (blue) or +20 mV (red). This stimulus primarily activated the RGC's receptive-field center, which has strong excitatory input and weak inhibitory input. **b**, Same as **a**, but the visual stimulus was an annulus with an inner diameter of 1000 µm and an outer diameter of 1200 µm. This stimulus primarily activated the RGC's receptive-field surround, which has weak excitatory input and strong inhibitory input. Source data are provided as a Source Data file.

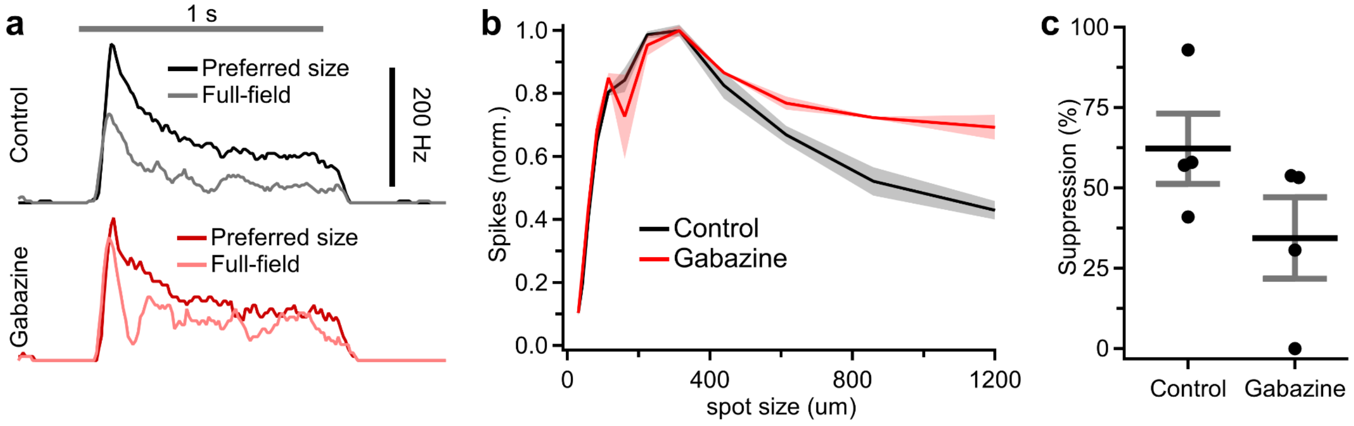

Supplementary Fig. 6 | Gabazine reduces surround suppression of Pix$_{ON}$ spiking response.
a, Example peristimulus time histograms recorded from a Pix$_{ON}$ RGC in response to preferred size (dark line) and full-field (lighter line) light spot stimuli in control conditions (bottom) and during bath application of Gabazine (10 μM). The gray horizontal bar indicates the 1-second presentation of the 250 R*/rod/s spot stimulus. **b**, Example spike rates recorded from a Pix$_{ON}$ RGC in response to a range of spot sizes in control conditions (black) and during bath application of Gabazine (red). **c**, Surround suppression of spiking responses in control and Gabazine conditions. Dots indicate data from individual Pix$_{ON}$ RGCs. Bar plots indicate average ± s.e.m.

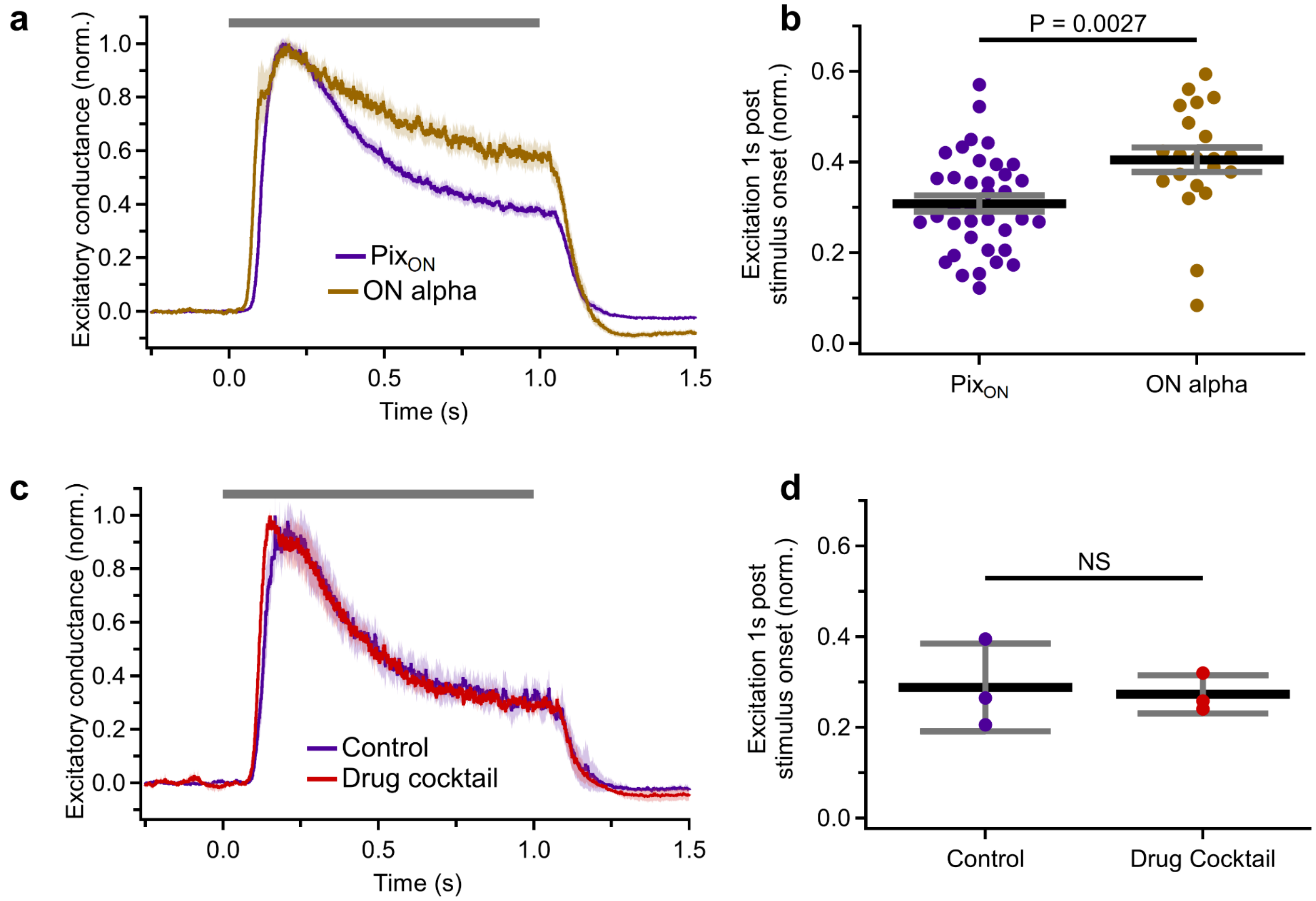

Supplementary Fig. 7 | Kinetics of excitatory conductances differ between Pix$_{ON}$ and ON alpha RGCs.
a, Average excitatory conductance of Pix$_{ON}$ RGCs (purple, n=37) and ON alpha RGCs (brown, n=21). Gray bar indicates 1s stimulation with a 220 µm diameter spot of light. Conductances from each cell were normalized by the maximum conductance value recorded for that cell. Shaded region indicates the standard error of the mean. **b**, The magnitude of the excitatory conductance 1s post-stimulus onset normalized as in **a**. Dots indicate individual Pix$_{ON}$ (purple, n=37) and ON alpha (brown, n=21) RGCs. The black bar indicates the average and gray bars indicate the standard error of the mean. **c**,**d**, Same as **a**,**b**, but comparing Pix$_{ON}$ excitatory conductances in control conditions (purple) to Pix$_{ON}$ excitatory conductances with bath application of a drug cocktail meant to block feedback from amacrine cells, nACh receptors, and NMDA receptors (strychnine, gabazine, saclofen, TPMPA, Hexamethonium, TTX, D-AP5; red, n=3).

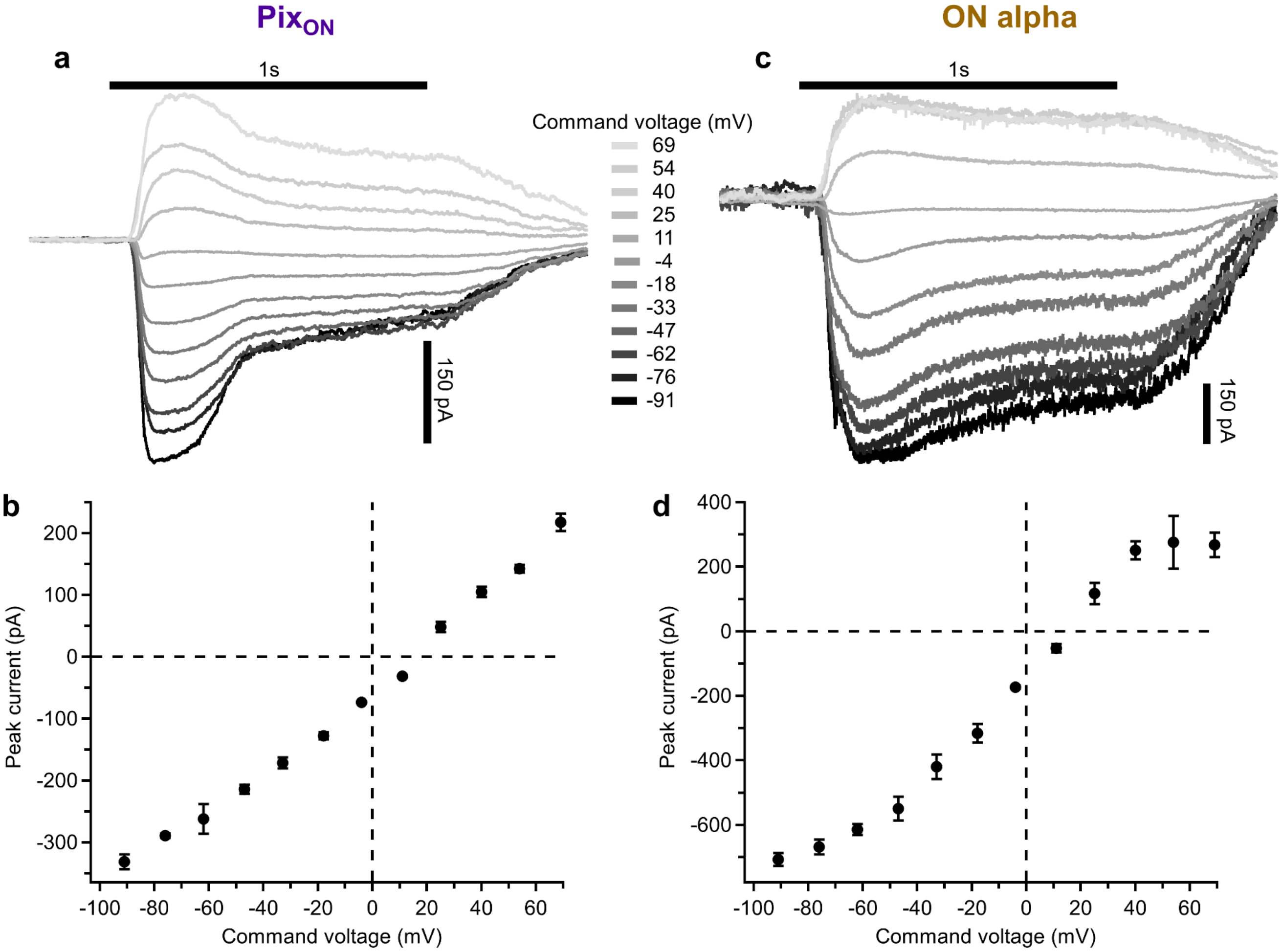

Supplementary Fig. 8 | Pix$_{ON}$ and ON alpha whole cell current-voltage (I-V) relationship suggests glutamatergic signaling is dominated by AMPA receptors.
a, Voltage-clamp recordings from a Pix$_{ON}$ RGC held at a range of command voltages. Currents are evoked by a 1s full-field light stimulus (horizontal black bar), during bath application of inhibitory blockers (gabazine, TPMPA, and strychnine). **b**, Relationship between command voltage and peak current recorded during the 1s light stimulus in **a**. Dots indicate average and bars indicate standard error across repeated trials (n=3). **c**,**d**, Same as **a**,**b**, but for recordings from an ON alpha RGC. Source data are provided as a Source Data file.

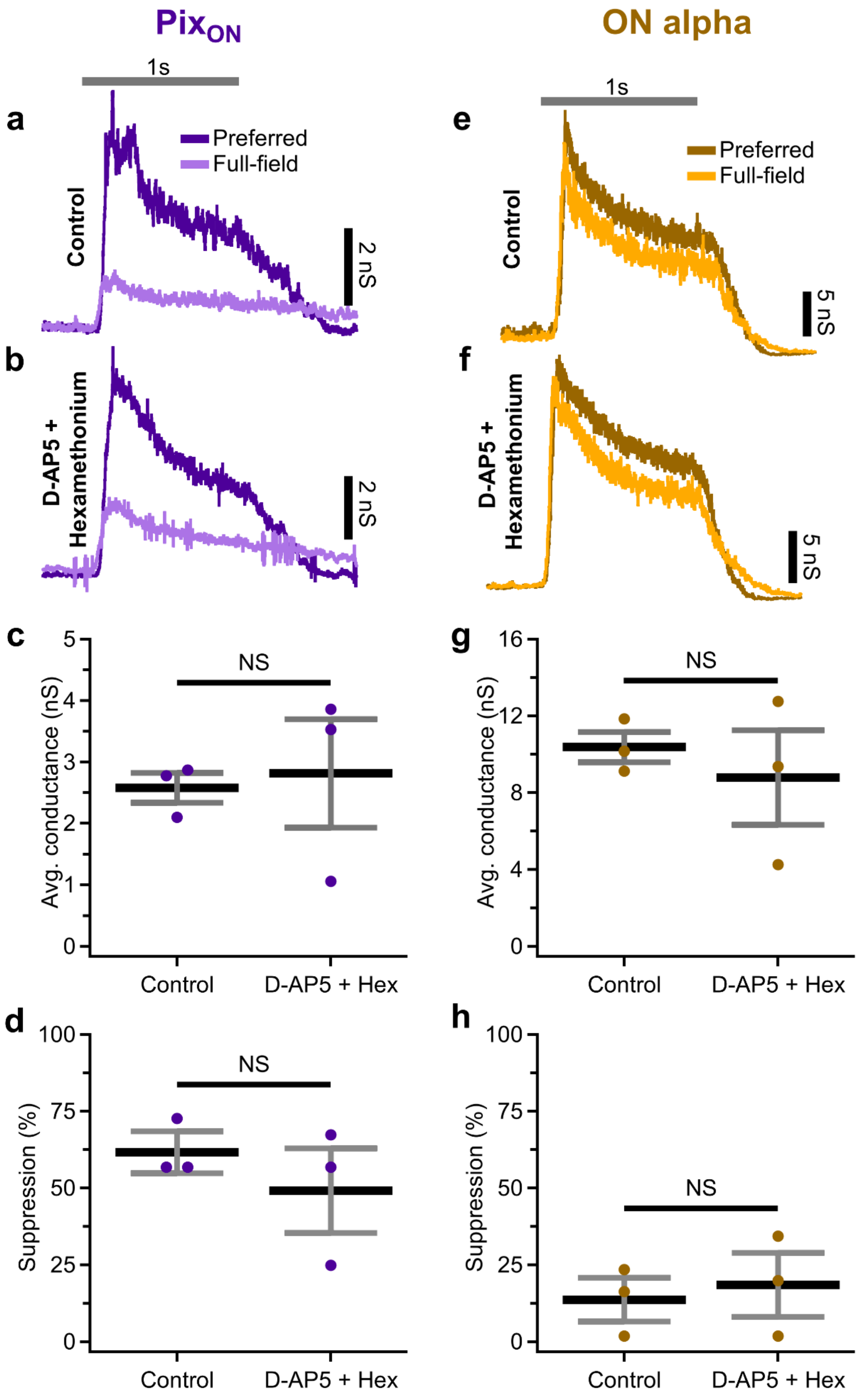

Supplementary Fig. 9 | Surround suppression of excitatory conductances during bath application of NMDA and nicotinic receptor antagonists.

a, Excitatory conductances recorded from a Pix_{ON} RGC during 1s stimulation of a preferred size or full-field stimulus. **b**, Same as **a**, but after bath application of D-AP5 (100 uM) and Hexamethonium (100 uM). **c**, Average excitatory conductance elicited by the preferred size stimuli in control conditions and during bath application D-AP5 + Hexamethonium for Pix_{ON} RGCs(n=3). **d**, Surround suppression of excitatory conductances recorded in control conditions and during bath application of D-AP5 + Hexamethonium for the Pix_{ON} RGC from **c**. **e**-**h**, Same as **a**-**d**, but recorded from ON alpha RGCs. Bar plots indicate average ± s.e.m., dots indicate individual RGCs, NS p>0.05, paired two-sample Student's t-test. Source data are provided as a Source Data file.

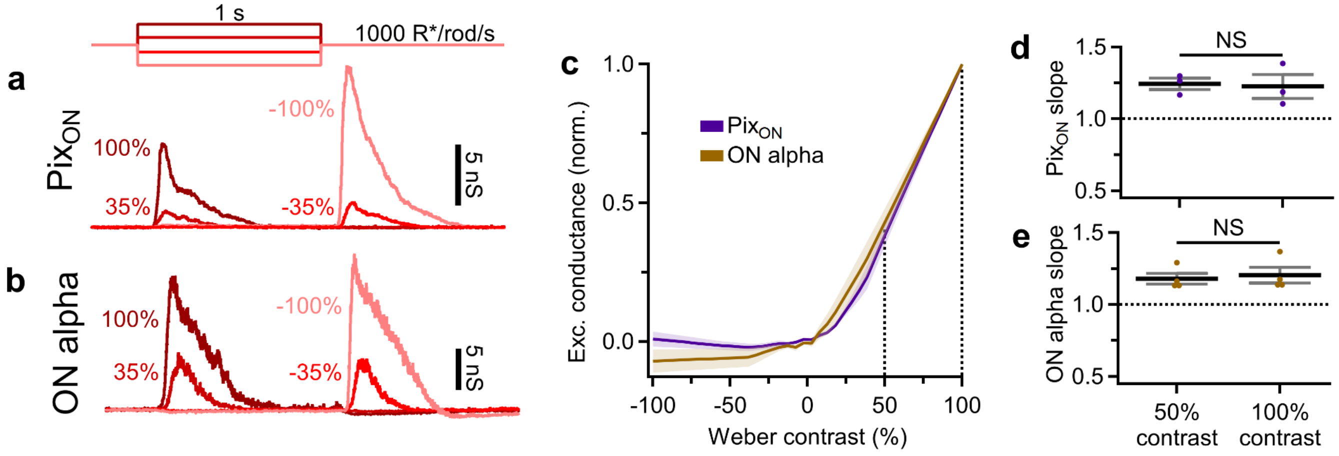

Supplementary Fig. 10 | Pix$_{ON}$ and ON alpha RGCs exhibit similar contrast response functions in their excitatory conductances.

a, Example Pix$_{ON}$ excitatory conductances evoked by stimulation of positive and negative contrast steps from a background illumination of 1000 rhodopsin isomerizations per rod per second (R*/rod/s). **b**, Same as **a**, but recorded from an ON alpha RGC. **c**, Excitatory responses measured across a range of Weber contrast steps for Pix$_{ON}$ (n=3) and ON alpha (n=3) RGCs. **d**, Pix$_{ON}$ contrast response slopes (Δ exc. nS / Δ contrast) are not different at 50% and 100% contrast, indicating that the Pix$_{ON}$ excitatory response has not begun saturating at 100% contrast compared to 50% contrast. **e**, Same as **d**, but for ON alpha RGCs. Dots indicate data from individual cells. Bar plots indicate average ± s.e.m., NS p>0.05, paired two-sample Student's *t*-test. Source data are provided as a Source Data file.

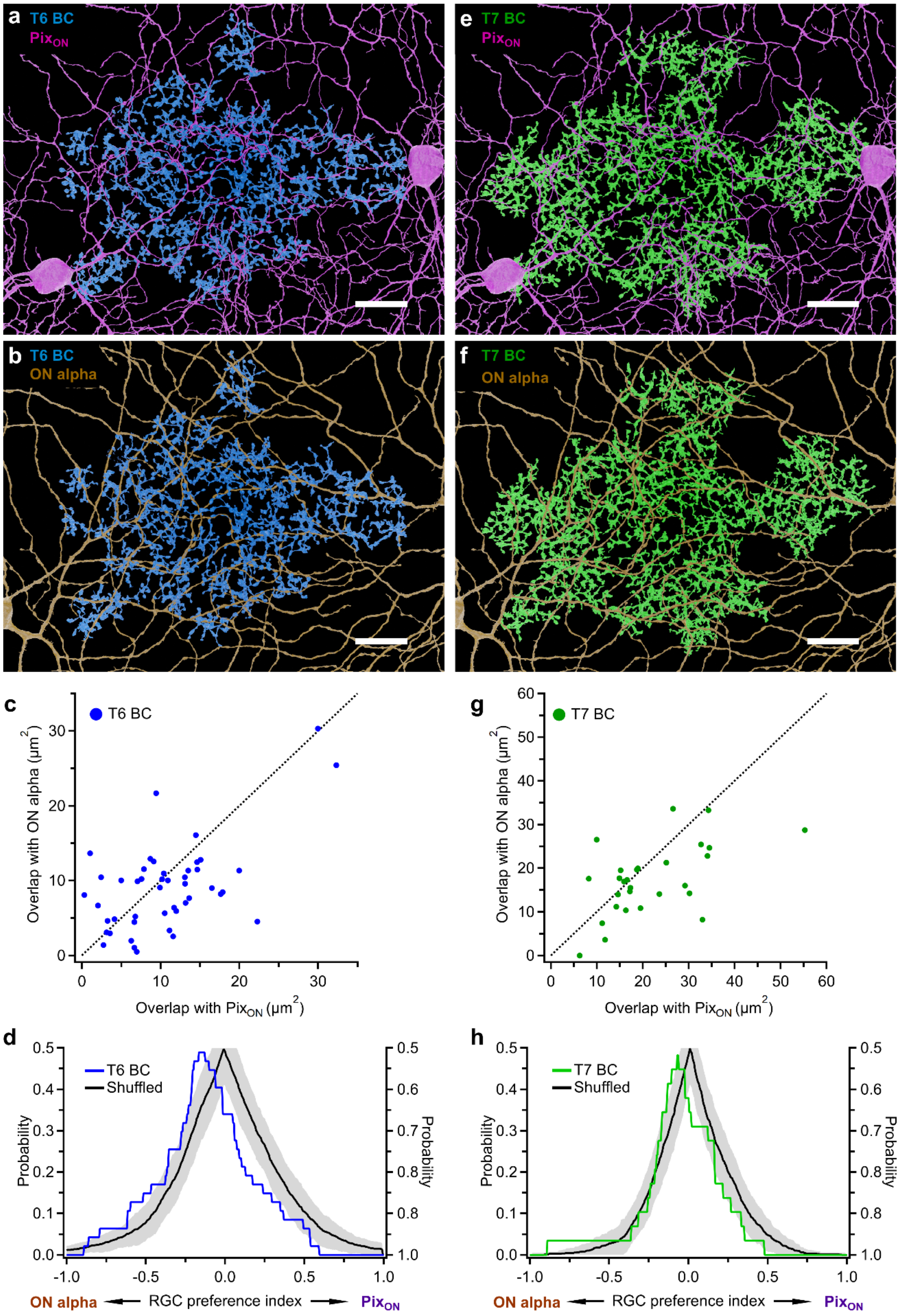

Supplementary Fig. 11 | Individual T6 and T7 BCs do not preferentially overlap with either Pix$_{ON}$ or ON alpha RGCs.
a, *En-face* view of 9n RGC (Pix$_{ON}$) dendrites and T6 BC axonal arbors from Eyewire reconstruction. **b**, same as **a**, but depicting 8w (ON alpha) RGC dendrites. **c**, Scatter plot showing the z-projection overlap for each T6 BC with Pix$_{ON}$ and ON alpha RGCs. Overlap was calculated as the total area each BC overlapped with either Pix$_{ON}$ or ON alpha RGCs as seen in an *en face* z-projection of their Eyewire reconstructions. **d**, Folded cumulative distribution (mountain plot) of T6 BC RGC preference index. RGC preference index was calculated for each BC as (Pix$_{ON}$ overlap - ON alpha overlap) / (Pix$_{ON}$ overlap + ON alpha overlap) with a value of 1 indicating a BC that only overlapped with Pix$_{ON}$ RGC dendrites and a value of -1 indicating a BC that only overlapped with ON alpha RGC dendrites. Shuffled indicates measurements obtained when randomly shifting each T6 BCs position in X and Y. **e**-**h**, same as **a**-**d**, but analyzing T7 BC arbor overlap with Pix$_{ON}$ and ON alpha dendrites. Source data are provided as a Source Data file.

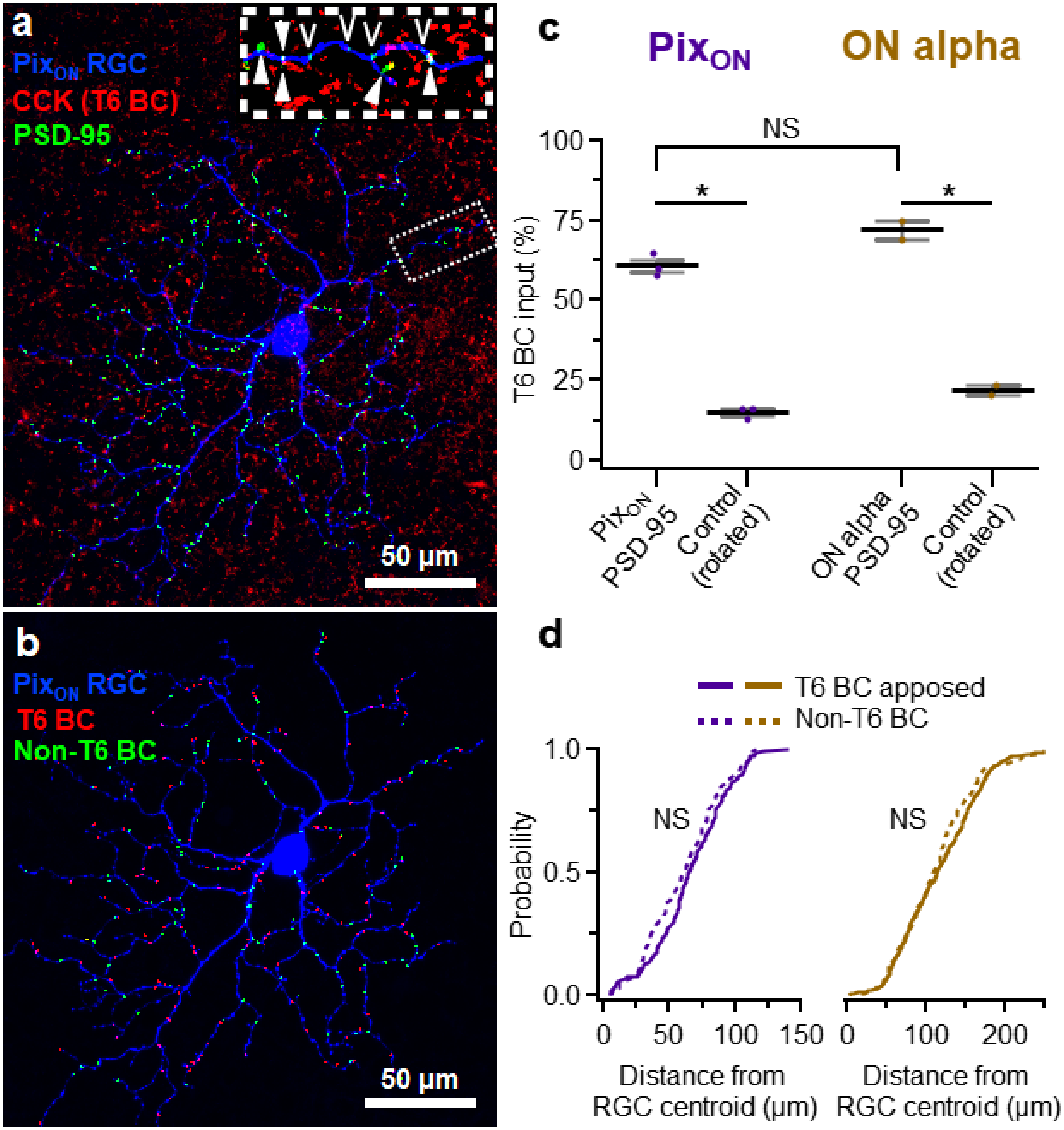

Supplementary Fig. 12 | PSD95 puncta apposed to type 6 bipolar cell terminals throughout the dendritic arbors of Pix_{ON} and ON alpha RGCs.

a, *En-face* view of a neurobiotin-filled Pix_{ON} RGC (blue) in the CCK-ires-Cre/Ai14 mouse line, which labels T6 BCs (red). Postsynaptic density protein 95 (PSD95; green) is immunohistochemically labeled to identify excitatory synapses on the RGC dendrite. Inset shows a zoomed-in view of the Pix_{ON} dendrite in which some PSD95 puncta are apposed to a T6 BC axon terminal (white closed arrow), while other PSD95 puncta are not apposed to a T6 BC axon terminal (white open arrow). **b**, Same Pix_{ON} RGC as in **a**, but all PSD95 puncta have been identified as apposed (red) or not-apposed (green) to a T6 BC terminal. **c**, Percentage of PSD95 puncta apposed to a T6 BC within the Pix_{ON} (n=3) and ON alpha (n=2) RGCs dendritic arbor. To estimate the chance probability of PSD95 puncta overlapping with T6 BC terminals, we performed a control analysis in which the PSD95 puncta image channel was rotated 90° compared to the T6 BC image channel. $^*p<0.05$, Welch's t-test was used for comparison of Pix_{ON} to ON alpha. Paired two-sample Student's *t*-test was used to compare the experimental group to a rotated control. **d**, Cumulative probability of distances between PSD95 puncta and the centroid of the RGC dendritic arbor, plotted for both T6 BC apposed and non-T6 BC apposed PSD95 puncta. Differing distributions of these distances would indicate that the proportion of T6 BC apposed and non-T6 BC apposed PSD95 vary by dendritic eccentricity. These distributions were not found to differ significantly for either Pix_{ON} (T6 n=163, non-T6 n=110) or ON alpha RGCs (T6 n=461, non-T6 n=157). p>.05, Kolmogorov–Smirnov test. Source data are provided as a Source Data file.

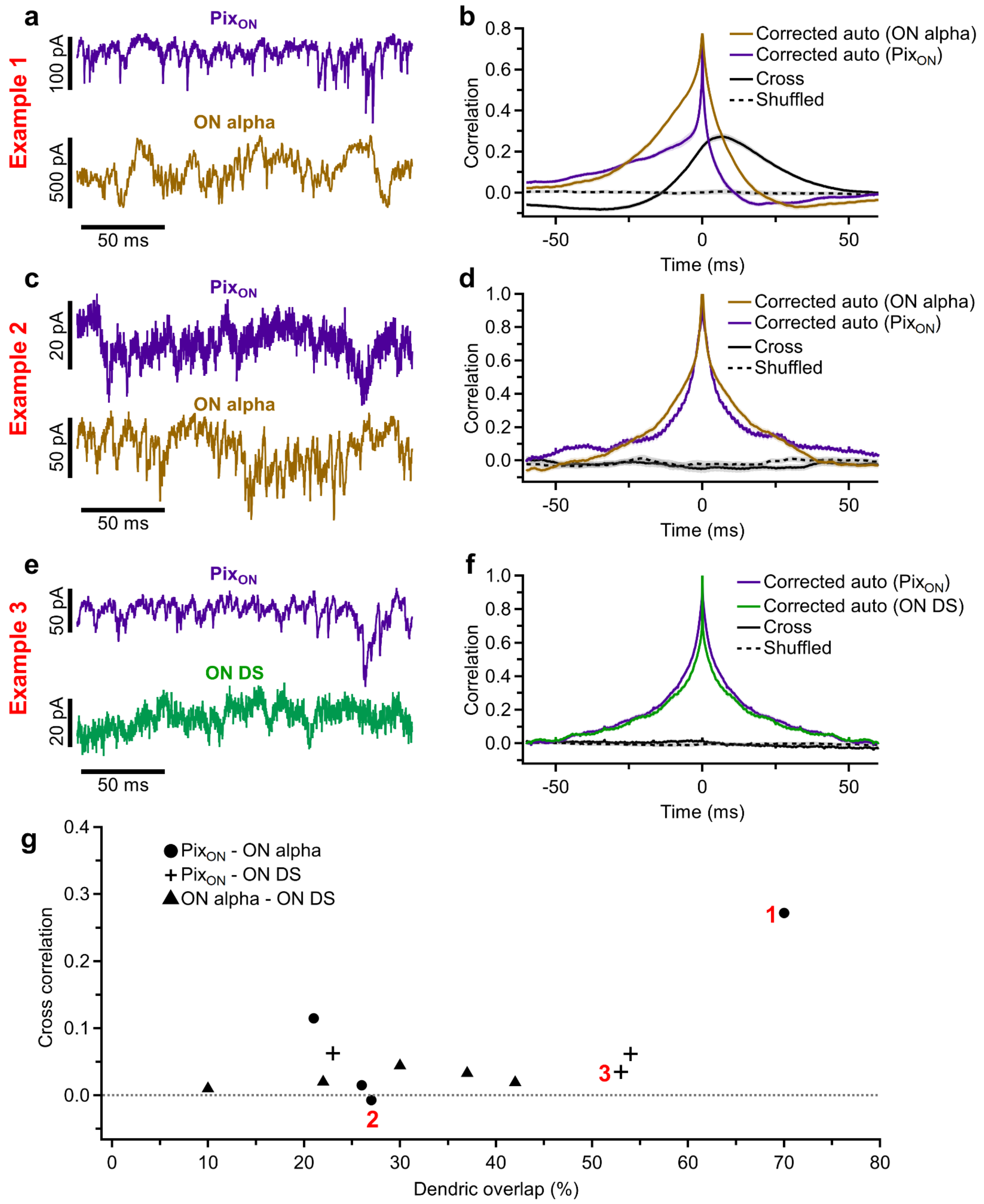

Supplementary Fig. 13 | Low noise correlation between Pix$_{ON}$ and ON alpha excitatory conductances.
a, Example paired voltage clamp recordings of excitatory conductances of a Pix$_{on}$ RGC (purple) and an ON alpha RGC (brown) which have overlapping dendritic arbors. **b**, Autocorrelation and cross-correlation of cells in **a**. Autocorrelation of the Pix$_{ON}$ (purple) and ON alpha (brown) were corrected for common noise by subtracting the cross-correlation (black). Dotted line indicates shuffled control of the cross-correlation. **c**,**d**, same as **a**,**b**, but for a Pix$_{ON}$ - ON alpha pair without correlated noise. **e**,**f**, Same as **a**,**b**, but for a control pair of cells (Pix$_{ON}$ and ON DS), which are expected to share photoreceptors, but not bipolar cells. **g**, Excitatory noise cross-correlation for Pix$_{ON}$-ON alpha RGC pairs (circles), Pix$_{ON}$ - ON DS RGC pairs (plus sign), and ON alpha - ON DS RGC pairs (triangles). Dendritic overlap indicates the area of the cells' dendric arbor which overlapped.

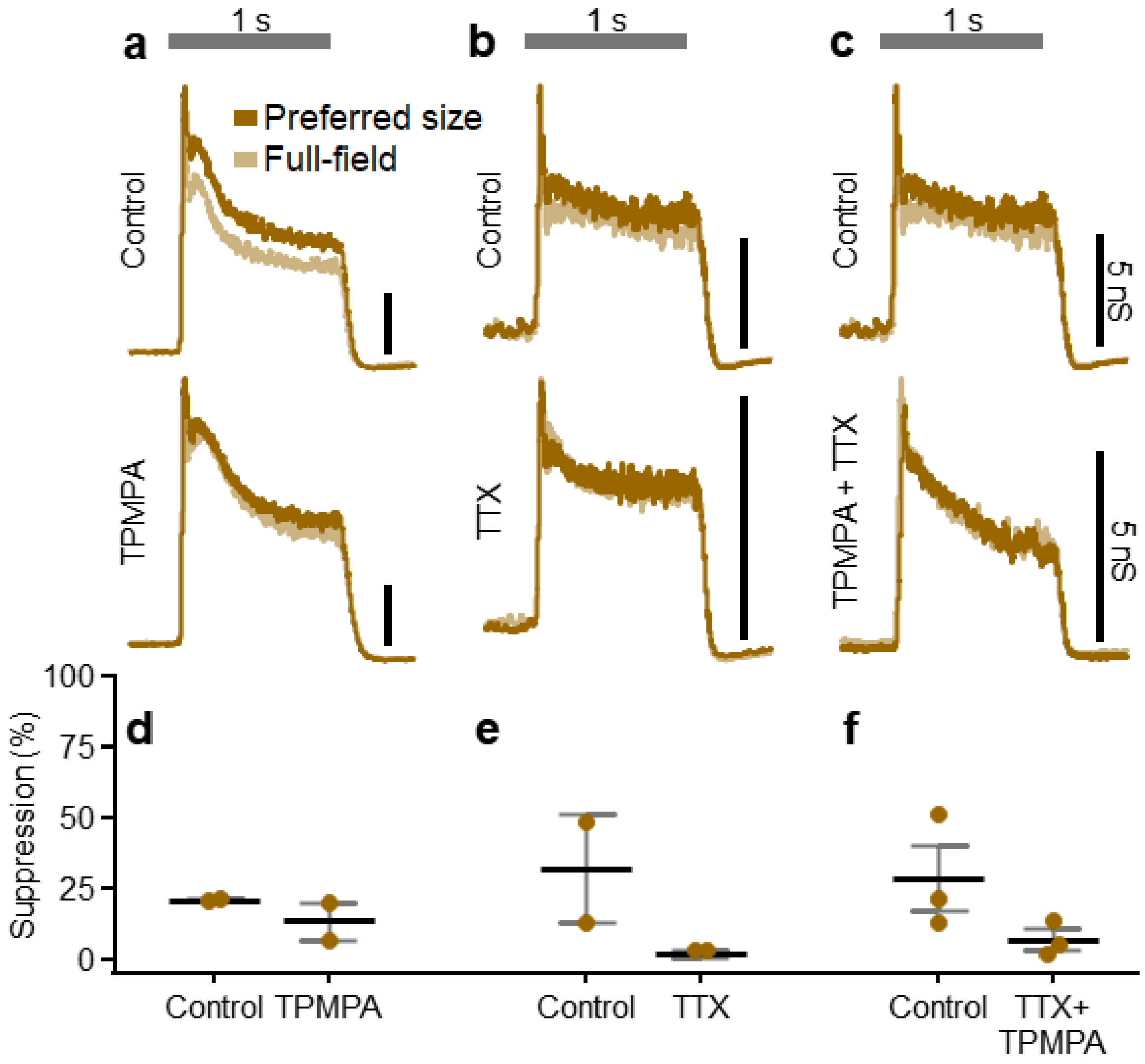

Supplementary Fig. 14 | ON alpha surround suppression of excitation remains weak in pharmacological block of $GABA_C$ receptors and Na_V channels.
a, ON alpha excitatory conductances evoked before (top) and after (bottom) bath application of the $GABA_C$ receptors antagonist TPMPA. The gray horizontal bar indicates a 1-second presentation of either the preferred size (dark brown) or full-field (light brown) spot stimuli. **b**, Same as **a**, but during bath application of the Na_V channel blocker TTX. **c**, Same as **a,** but during dual application of TPMPA and TTX. **d**, Surround suppression of ON alpha excitatory conductances in control conditions and during bath application of TPMPA (n=3). **e**, Same as **d**, but during bath application of TTX (n=2). **f**, Same as **d**, but during dual application of TPMPA and TTX (n=3). **d-f**, Dots indicate data from individual cells. Bar plots indicate average ± s.e.m. Source data are provided as a Source Data file.

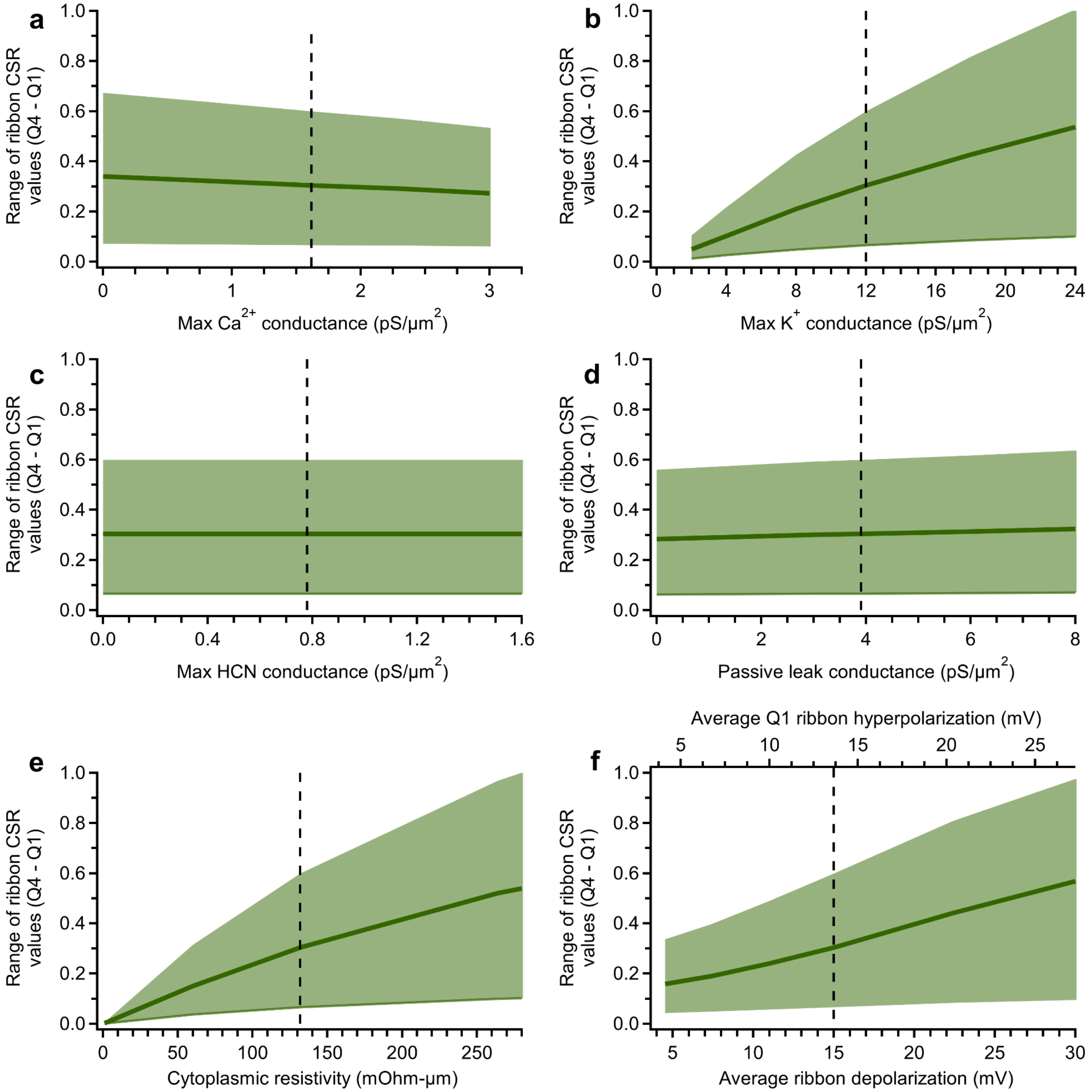

Supplementary Fig. 15 | Cable model consistency over a range of parameter values.
a-f, Range of CSR values resulting when varying model parameter values (see **Fig. 7**). Simulations were performed when activating 60 N-nearest inhibitory synapses. Thick lines indicate the median decay across all sets of inhibitory synapses activated. Shading indicates the range (maximum to minimum) of CSR values recorded when activating each of the 120 sets of 60 inhibitory synapses. The vertical dotted line indicates the parameter value used in all other simulations. **b**, Note: Simulations could not be performed with zero K^+ conductance, as Ca^{2+} currents would cause uncontrolled depolarization without some counterbalancing K^+ currents. **f**, Ribbon depolarization and hyperpolarization had to be increased simultaneously to maintain the same center and surround ratio for the first quartile of ribbon synapses. Otherwise, the nonlinear nature of CSR interactions would skew results (eg. higher overall CSR values lead to a greater CSR range). Source data are provided as a Source Data file.

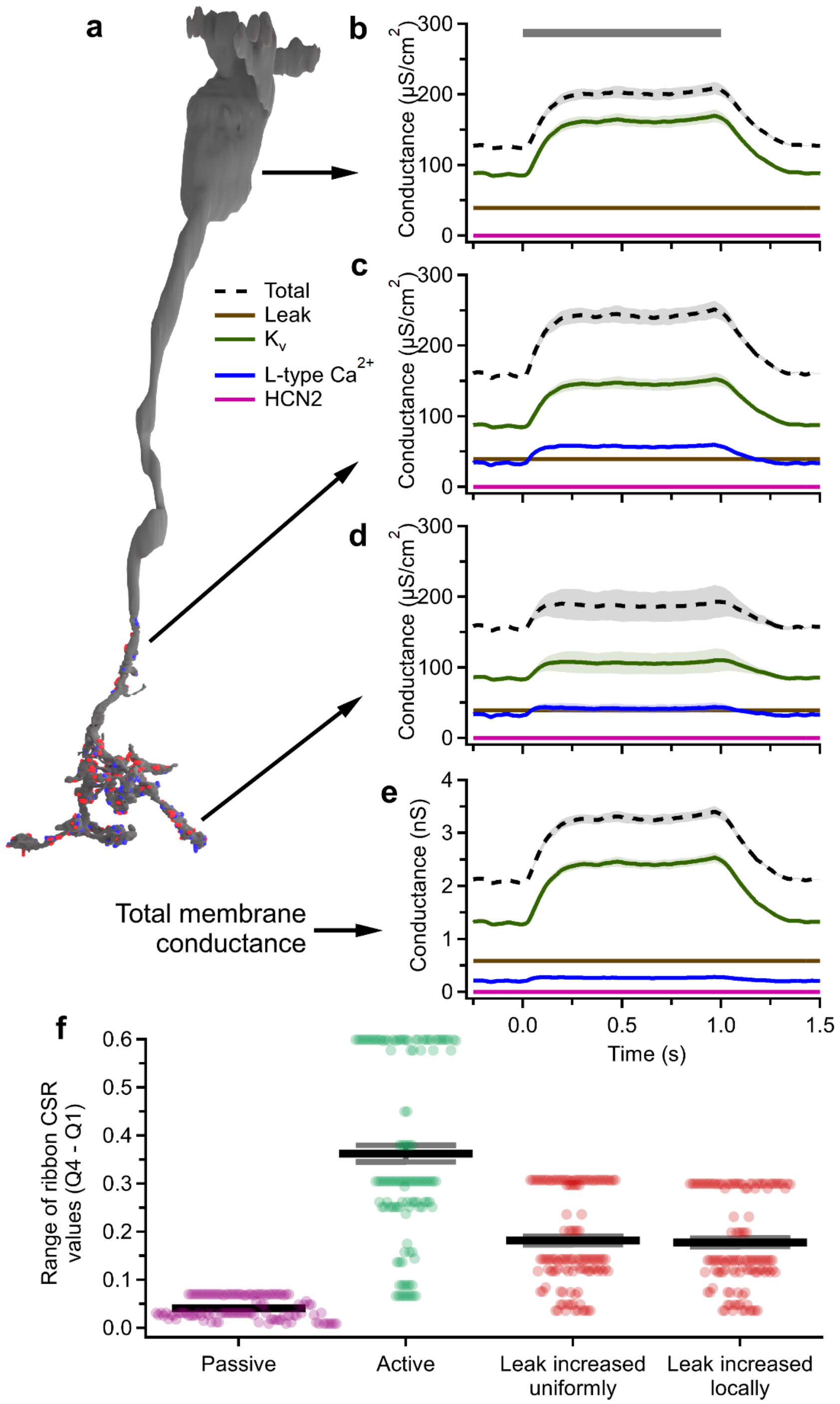

Supplementary Fig. 16 | Increasing leak conductance increases the heterogeneity of ribbon CSR values.
a, Cable model of a T6 BC (as shown in **Fig. 7**). **b**, Conductances of the active and passive membrane channels located at the cell's soma during a simulation in which 60 inhibitory synapses were simultaneously activated for 1 s (gray bar). The thick line indicates the mean, and the shaded region indicates the standard deviation obtained when activating each of the 120 sets of 60 inhibitory synapses. **c,** Same as **b** but for membrane channels located on the axonal stalk prior to the first branching point. **d**, Same as **b**, but for membrane channels located at a terminal point of the axon. **e**, Same as **b**, but for total conductances summed across the cell's membrane. **f**, Range of CSR values when modifying membrane conductances in various ways. "Passive" and "Active" refer to the passive and active models shown in **Figure 7d**. "Leak increased uniformly" indicates a model without any active conductances but in which the passive leak conductance is increased uniformly across the entire cell membrane to match the average total membrane conductance of the active model during the stimulation period (see **e**). "Leak increased locally" indicates a model without any active conductances but in which the passive leak conductance is increased in each of the model compartments to match the average total conductance measured in the same compartment of the active model during the stimulation period (see **b**-**d**). Source data are provided as a Source Data file.

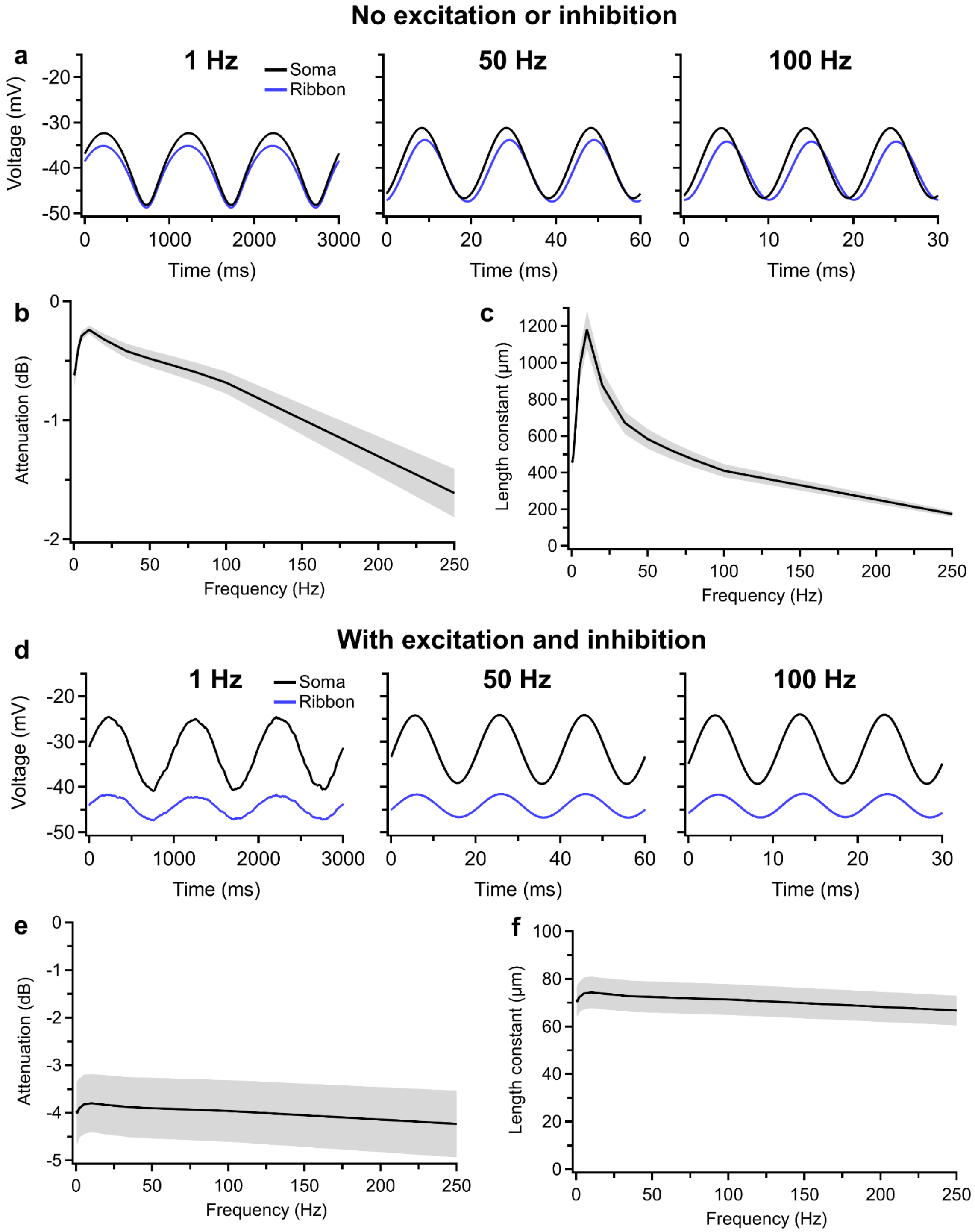

Supplementary Fig. 17 | Electrical compactness across frequency ranges of stimulation.
a, Voltage recorded at the soma (*black*) and at an example ribbon output synapse (*blue*) in response to sinusoidal current injection at the soma, with sinusoidal frequencies of 1Hz (*left*), 50 Hz (*middle*), and 100 Hz (*right*). **b**, Attenuation of voltage signal (RMS) recorded at ribbon output synapses compared to the voltage signal (RMS) at the soma. **c**, Length constant calculated for each ribbon as a function of voltage attenuation and path distance from the soma (see **methods**). **a**-**c**, Note: These simulations were performed without activating excitatory or inhibitory synapses. To maintain the same average membrane voltage (-38 mV) as produced in previous simulations which activated excitatory and inhibitory synapses (**Fig. 7**), a constant 37 pA was injected at the soma. **d-f**, Same as **a-c**, but excitatory and inhibitory synapses were activated in the same manner as **Fig. 7**. Note: DC current injections were no longer required to maintain consistency with previous simulations. **b**,**c**,**e**,**f**, Thick line indicates the average ribbon synapse attenuation, and the shaded region indicates the standard deviation of attenuation across the 84 ribbon synapses. All simulations were performed in the active model of the T6 BC (includes L-type Ca^{2+} channels, K_V^+ channels, and HCN_2 channels). Source data are provided as a Source Data file.

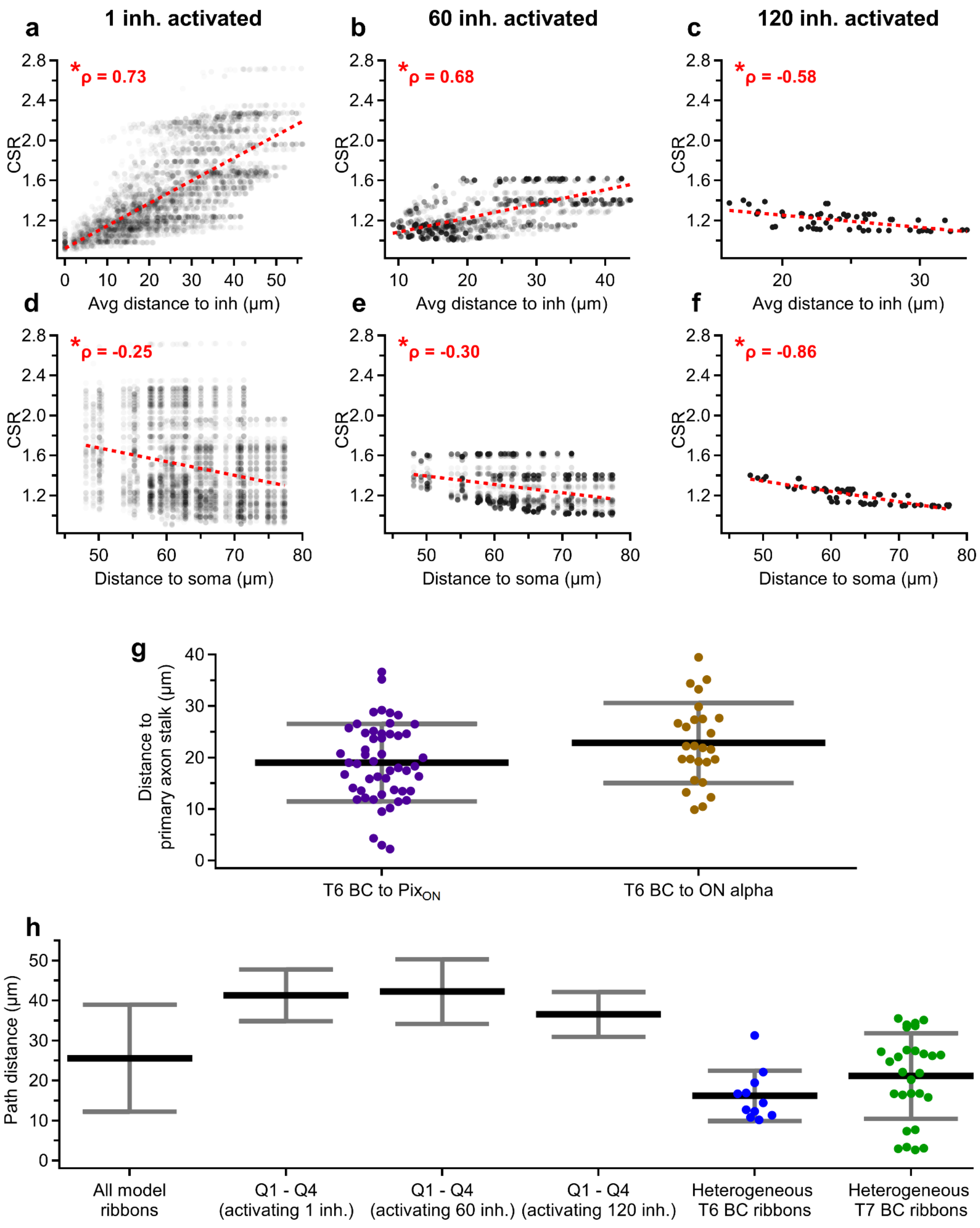

Supplementary Fig. 18 | Comparing anatomical correlates of model results to SBFSEM reconstructions.
a, CSR values measured at each ribbon synapse plotted against their path distance to the single activated inhibitory synapse (see **Fig. 7**). Red dotted line indicates a linear fit to the data. "ρ" indicates Pearson's Linear Correlation Coefficient and "*" indicates p<0.01. **b**, Same as **a**, but sets of 60 N-nearest inhibitory synapses are activated, and the average path distance to these 60 activated inhibitory is calculated for each ribbon synapse. **c**, Same as **b**, but all 120 inhibitory synapses are activated. **d-f**, CSR values measured at each ribbon synapse plotted against their path distance to the soma when activating sets of 1 (**d**), 60 (**e**), or 120 (**f**) N-nearest inhibitory synapses. **g**, Path distance to primary axon stalk (first branching point of the axon) for ribbon synapses onto Pix$_{ON}$ RGCs and ON alpha RGCs as measured from SBFSEM (see **Fig. 5**). Dots indicate measurements taken from individual ribbon synapses that synapse onto each RGC type (Pix$_{ON}$ n = 51, ON alpha n = 26). **h**, Average path distance between ribbon synapses. "All model ribbons" refers to the pairwise distances between all 84 ribbon synapses in the T6 BC model. "Q1 - Q4" refers to the pairwise path distances between the ribbons with the highest quartile of CSR values and the ribbons with the lowest quartile of CSR values. Measurements were taken for model simulations in which one inhibitory synapse was activated (activating 1 inh.), when 60 inhibitory synapses were activated (activating 60 inh.), or when all 120 inhibitory synapses were activated (activating 120 inh.). "Heterogeneous T6 BC ribbons" refers to the path distances measured from the SBFSEM reconstruction between T6-Pix$_{ON}$ synapses and T6-ON alpha synapses within the same T6 BC (see **Fig. 5**). "Heterogeneous T7 BC ribbons" refers to the path distance measured from the SBFSEM reconstruction between T7-Pix$_{ON}$ synapses and T7-ON alpha synapses within the same T7 BC. **a-f**,**h,** All CSR values are obtained from simulations in the active model of the T6 BC. Source data are provided as a Source Data file.

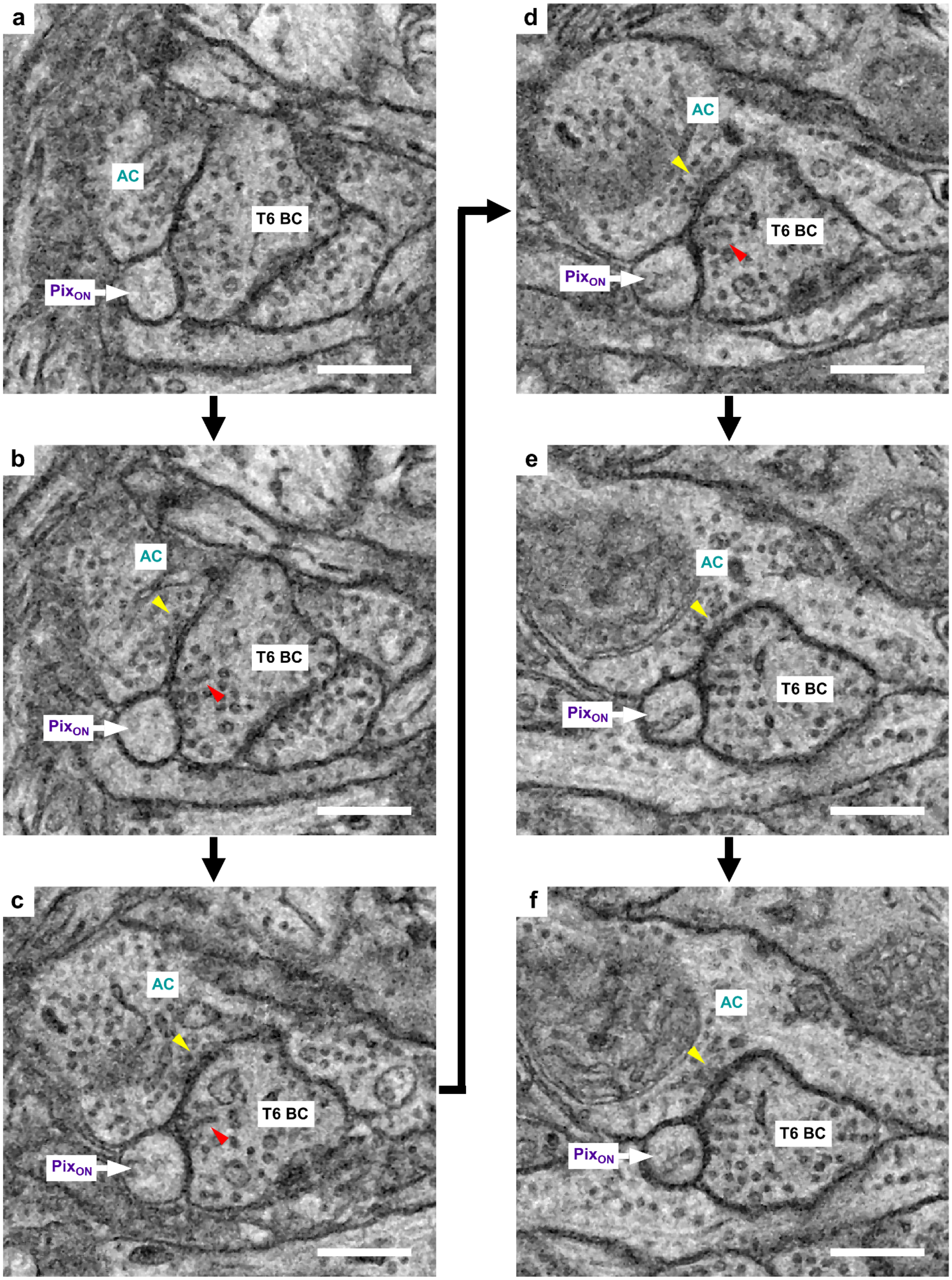

Supplementary Fig. 19 | Identification of synapses across SBFSEM z-sections.
a-**f**, SBFSEM slices showing an AC neurite forming an inhibitory synapse (yellow arrow) onto a T6 BC, which then forms a ribbon synapse (red arrow) onto a Pix$_{ON}$ RGC dendrite. Each image depicts the same XY location (same as **Fig. 6c**), with sequential images progressing 50 nm deeper into the IPL. The white scale bar is 500 nm.

Supplementary Table 1 | Pharmacological agents			
**Pharmacological agent**	**Target**	**Concentration**	**Vendor**
Tetrodotoxin (TTX)	Voltage-gated sodium channel blocker[1]	500 nM	Tocris #1078
Gabazine (SR-95531)	$GABA_A$ receptor antagonist[2]	10 μM	Sigma #S106
TPMPA ((1,2,5,6-Tetrahydropyridin-4-yl)methylphosphinic acid)	$GABA_C$ receptor antagonist[3]	50 μM	Tocris #1040
Saclofen (3-Amino-2-(4-chlorophenyl)-2-hydroxypropanesulfonic acid)	$GABA_B$ receptor antagonist[4]	100 μM	Sigma #S116
Strychnine Hydrochloride	glycine receptor antagonis[5]	1 μM	Sigma #S8753
Kynurenic acid (4-Hydroxyquinoline-2-carboxylic acid)	Weak AMPA, kainate, and NMDA receptor antagonist[6]	700 nM	Sigma #K3375
NBQX (2,3-dioxo-6-nitro-7-sulfamoyl-benzo[f]quinoxaline)	Strong AMPA and kainate receptor antagonist[7]	300 nM	Tocris #1044
L-AP4 (L-(+)-2-Amino-4-phosphonobutyric acid)	group III mGluR agonist[8]	100 μM	Tocris #0103
DNQX (6,7-Dinitroquinoxaline-2,3-dione disodium salt)	non-NMDA iGluR antagonist[9]	40 μM	Tocris #2312
D-AP5 (D-(-)-2-Amino-5-phosphonopentanoic acid)	NMDA receptor antagonist[10]	30 μM	Tocris #0106

Supplementary Table 2 | Antibodies used in immunohistochemical labeling			
**Primary antibody**		**Secondary antibody**	
**Name**	**Vendor**	**Name**	**Vendor**
Rabbit anti-PSD95	Cell Signaling #3450	Donkey anti-rabbit Alexa Fluor 647	Jackson Immuno #711-605-152
Goat anti-ChAT	Millipore #AB144P	Donkey anti-goat Alexa Fluor 647	Life Technologies #A21447
Mouse anti-SMI-32	BioLegend #801702	Donkey anti-mouse Alexa Fluor 647	Life Technologies #A31571
		Streptavidin 488	Thermo Science #21832

**Supplementary Table 3 | Key parameters of T6 BC NEURON model**		
Experimental parameters	Time step	25 μs
	Temperature	32°
Passive properties	Membrane capacitance	11.8 fF/μm^2 [ref.[11]]
	Cytoplasmic resistivity	13.2 mOhm-μm [ref.[11]]
	Leak conductance	3.9 pS/μm^2 [ref.[11]]
	Leak reversal potential	-60 mV [ref.[11]]
Excitation at dendrites (8 synapses)	Reversal potential	10.1 mV [ref.[12]]
	Rise time	10 ms [ref.[13]]
	Decay time	100 ms [ref.[13]]
Inhibition at axon ($GABA_C$)	Reversal potential	−50.4 mV [ref.[14]]
	Rise time	1.8 ms [ref.[15]]
	Decay time	100 ms [ref.[15]]
HCN2 channel (restricted to axonal arbor) [ref.[16,17]]	Reversal potential	-23.4 mV[18]
	Max conductance	0.78 pS/μm^2 [ref.[16,17]]
	Half-max of activation	-99 mV [ref.[19]]
	Slope of activation	-6.2 mV [ref.[19]]
K_V^+ channel [ref.[20]]	K^+ equilibrium potential	-84 mV
	Max conductance	12 pS/μm^2 [ref.[20]]
	Half-max of activation	-9 mV [ref.[21]]
	Slope of activation	14 mV [ref.[21]]
	Half-max of inactivation	8 mV [ref.[21]]
	Slope of inactivation	-9 mV [ref.[21]]
L-type Ca^{2+} channels (restricted to axonal arbor) [ref.[22]]	Ca^{2+} reversal potential	18 mV [ref.[22]]
	L-type Ca^{2+} max conductance	1.6 pS/μm^2 [ref.[22]]
	L-type Ca^{2+} half-max activation	-32 mV [ref.[22]]
	L-type Ca^{2+} slope of activation	10 mV [ref.[22]]
	L-type Ca^{2+} half-max inactivation	10 mV [ref.[22]]
	L-type Ca^{2+} slope of inactivation	-12 mV [ref.[22]]

Supplemental References

1. Narahashi, T., Moore, J. W. & Scott, W. R. Tetrodotoxin Blockage of Sodium Conductance Increase in Lobster Giant Axons. *Journal of General Physiology* vol. 47 965–974 Preprint at https://doi.org/10.1085/jgp.47.5.965 (1964).
2. Ueno, S., Bracamontes, J., Zorumski, C., Weiss, D. S. & Steinbach, J. H. Bicuculline and Gabazine Are Allosteric Inhibitors of Channel Opening of the $GABA_A$Receptor. *The Journal of Neuroscience* vol. 17 625–634 Preprint at https://doi.org/10.1523/jneurosci.17-02-00625.1997 (1997).
3. Li, S., Zhang, Y., Liu, H., Yan, Y. & Li, Y. Identification and expression of $GABA_C$receptor in rat testis and spermatozoa. *Acta Biochimica et Biophysica Sinica* vol. 40 761–767 Preprint at https://doi.org/10.1111/j.1745-7270.2008.00453.x (2008).
4. Kerr, D. I. B. *et al.* GABAB receptor antagonism by resolved (R)-saclofen in the guinea-pig ileum. *European Journal of Pharmacology* vol. 308 R1–R2 Preprint at https://doi.org/10.1016/0014-2999(96)00334-2 (1996).
5. O’connor, V. INTERACTIONS OF GLYCINE AND STRYCHNINE WITH THEIR RECEPTOR RECOGNITION SITES IN MOUSE SPINAL CORD. *Neurochemistry International* vol. 29 423–434 Preprint at https://doi.org/10.1016/0197-0186(95)00160-3 (1996).
6. Weber, M. *et al.* 6-Hydroxykynurenic acid and kynurenic acid differently antagonise AMPA and NMDA receptors in hippocampal neurones. *J. Neurochem.* **77**, 1108–1115 (2001).
7. Kovács, I. *et al.* Cyclothiazide binding to functionally active AMPA receptor reveals genuine allosteric interaction with agonist binding sites. *Neurochem. Int.* **44**, 271–280 (2004).
8. Bushell, T. J. *et al.* Antagonism of the synaptic depressant actions of L-AP4 in the lateral perforant path by MAP4. *Neuropharmacology* **34**, 239–241 (1995).
9. Honoré, T. *et al.* Quinoxalinediones: potent competitive non-NMDA glutamate receptor antagonists. *Science* **241**, 701–703 (1988).
10. Davies, J. & Watkins, J. C. Actions of D and L forms of 2-amino-5-phosphonovalerate and 2-amino-4-phosphonobutyrate in the cat spinal cord. *Brain Res.* **235**, 378–386 (1982).
11. Oltedal, L., Veruki, M. L. & Hartveit, E. Passive membrane properties and electrotonic signal processing in retinal rod bipolar cells. *J. Physiol.* **587**, 829–849 (2009).

12. Nawy, S. Regulation of the on bipolar cell mGluR6 pathway by Ca2+. *Journal of Neuroscience* (2000).
13. Kukaj, T., Sattler, C., Zimmer, T., Schmauder, R. & Benndorf, K. Kinetic fingerprinting of metabotropic glutamate receptors. *Commun Biol* **6**, 104 (2023).
14. Billups, D. & Attwell, D. Control of intracellular chloride concentration and GABA response polarity in rat retinal ON bipolar cells. *J. Physiol.* (2002).
15. Schubert, T. *et al.* Development of presynaptic inhibition onto retinal bipolar cell axon terminals is subclass-specific. *J. Neurophysiol.* **100**, 304–316 (2008).
16. Ivanova, E. & Müller, F. Retinal bipolar cell types differ in their inventory of ion channels. *Vis. Neurosci.* **23**, 143–154 (2006).
17. Müller, F. *et al.* HCN channels are expressed differentially in retinal bipolar cells and concentrated at synaptic terminals. *Eur. J. Neurosci.* **17**, 2084–2096 (2003).
18. Byczkowicz, N. *et al.* HCN channel-mediated neuromodulation can control action potential velocity and fidelity in central axons. *Elife* **8**, (2019).
19. Moosmang, S. *et al.* Cellular expression and functional characterization of four hyperpolarization-activated pacemaker channels in cardiac and neuronal tissues. *Eur. J. Biochem.* **268**, 1646–1652 (2001).
20. Hu, H.-J. & Pan, Z.-H. Differential expression of K+ currents in mammalian retinal bipolar cells. *Vis. Neurosci.* **19**, 163–173 (2002).
21. Sprunger, L. K., Stewig, N. J. & O'Grady, S. M. Effects of charybdotoxin on K+ channel (KV1.2) deactivation and inactivation kinetics. *Eur. J. Pharmacol.* **314**, 357–364 (1996).
22. Berntson, A., Rowland Taylor, W. & Morgans, C. W. Molecular identity, synaptic localization, and physiology of calcium channels in retinal bipolar cells. *Journal of Neuroscience Research* vol. 71 146–151 Preprint at https://doi.org/10.1002/jnr.10459 (2003).
