# Supplementary material for: A presynaptic source drives differing levels of surround suppression in two mouse retinal ganglion cell types
Source: Nat Commun. 2024 Jan 18;15:599. doi: 10.1038/s41467-024-44851-w (PMC10796971; doi:10.1038/s41467-024-44851-w)
Supplement: Supplementary file 3 — Reporting Summary [file 41467_2024_44851_MOESM3_ESM.pdf]

nature portfolio

Corresponding author(s): Greg Schwartz

Last updated by author(s): 12-10-2023

Reporting Summary

Nature Portfolio wishes to improve the reproducibility of the work that we publish. This form provides structure for consistency and transparency in reporting. For further information on Nature Portfolio policies, see our Editorial Policies and the Editorial Policy Checklist.

Statistics

For all statistical analyses, confirm that the following items are present in the figure legend, table legend, main text, or Methods section.

n/a	Confirmed	
☐	☒	The exact sample size (n) for each experimental group/condition, given as a discrete number and unit of measurement
☐	☒	A statement on whether measurements were taken from distinct samples or whether the same sample was measured repeatedly
☐	☒	The statistical test(s) used AND whether they are one- or two-sided<br>*Only common tests should be described solely by name; describe more complex techniques in the Methods section.*
☐	☒	A description of all covariates tested
☐	☒	A description of any assumptions or corrections, such as tests of normality and adjustment for multiple comparisons
☐	☒	A full description of the statistical parameters including central tendency (e.g. means) or other basic estimates (e.g. regression coefficient) AND variation (e.g. standard deviation) or associated estimates of uncertainty (e.g. confidence intervals)
☐	☒	For null hypothesis testing, the test statistic (e.g. F, t, r) with confidence intervals, effect sizes, degrees of freedom and P value noted<br>*Give P values as exact values whenever suitable.*
☒	☐	For Bayesian analysis, information on the choice of priors and Markov chain Monte Carlo settings
☒	☐	For hierarchical and complex designs, identification of the appropriate level for tests and full reporting of outcomes
☐	☒	Estimates of effect sizes (e.g. Cohen's d, Pearson's r), indicating how they were calculated

Our web collection on statistics for biologists contains articles on many of the points above.

Software and code

Policy information about availability of computer code

Data collection	Physiology data was collected using MATLAB 2018b using custom code available at:<br>https://github.com/Schwartz-AlaLaurila-Labs/sa-labs-extension
Data analysis	Image analysis was performed with FIJI version 1.53t<br>Cells skeletons were traced with the FIJI plugin SNT 4.1.10<br>Physiology data was analyzed with MATLAB 2019b and custom code available at https://github.com/SchwartzNU/SymphonyAnalysis<br>Bipolar cell biophysical simulations were performed with Python 3.8 and NEURON 8.0.<br>Custom code for the bipolar cell receptive field model can be found at https://github.com/davidswygart/rgc_bipolar_dog<br>Custom code for the bipolar cell cable model can be found at https://github.com/davidswygart/T6_NEURON_python

For manuscripts utilizing custom algorithms or software that are central to the research but not yet described in published literature, software must be made available to editors and reviewers. We strongly encourage code deposition in a community repository (e.g. GitHub). See the Nature Portfolio guidelines for submitting code & software for further information.

Data

Policy information about availability of data

All manuscripts must include a data availability statement. This statement should provide the following information, where applicable:

- Accession codes, unique identifiers, or web links for publicly available datasets
- A description of any restrictions on data availability
- For clinical datasets or third party data, please ensure that the statement adheres to our policy

Data for the bipolar cell receptive field model can be found at https://github.com/davidswygart/rgc_bipolar_dog
Data for the bipolar cell cable model can be found at https://github.com/davidswygart/T6_NEURON_python
All other data will be posted on data.mendeley.com prior to final submission.

Research involving human participants, their data, or biological material

Policy information about studies with human participants or human data. See also policy information about sex, gender (identity/presentation), and sexual orientation and race, ethnicity and racism.

Reporting on sex and gender	No Human research participants were used.
Reporting on race, ethnicity, or other socially relevant groupings	No Human research participants were used.
Population characteristics	No Human research participants were used.
Recruitment	No Human research participants were used.
Ethics oversight	No Human research participants were used.

Note that full information on the approval of the study protocol must also be provided in the manuscript.

Field-specific reporting

Please select the one below that is the best fit for your research. If you are not sure, read the appropriate sections before making your selection.

☒ Life sciences ☐ Behavioural & social sciences ☐ Ecological, evolutionary & environmental sciences

For a reference copy of the document with all sections, see nature.com/documents/nr-reporting-summary-flat.pdf

Life sciences study design

All studies must disclose on these points even when the disclosure is negative.

Sample size	Physiology Data: A high N had already been collected in control conditions measuring RGC spiking output across stimulus spot sizes before surround suppression was calculated (PixON n = 46; ON alpha n = 90). This data showed an average difference of in surround suppression between the two cell types 63%, which was found to be highly significant p=10^-47. Subsequent experiments investigating the source of this differing surround suppression were conducted with an n such that surround suppression could be determined to within 7% (+- SEM), which would test surround suppression of similar magnitude to control conditions with a high degree of certainty.
Data exclusions	Prior to calculating surround suppression, PixON RGCs with spiking responses of less than 50 spikes over a 1 second stimulation of an optimal spot size were discarded as they were considered poor light responses. Likewise, ON alpha RGCs with less than 150 spikes over a 1 second simulation were discarded.
Replication	Each experiment was performed once. However, multiple experiments were performed to test the same hypothesis and the robustness of our interpretation of the data. Measures of bipolar type input were performed using EM and IHC with IHC. Surround suppression was measured in scotopic and photopic conditions and at multiple retinal locations. Model simulations were performed across a wide range of parameters.
Randomization	Mice within the chosen age range (6-36 weeks) were randomly chosen for experiments. RGCs were randomly targeted. Experimental groups were dependent upon RGC type, not mouse type, so experimental comparisons could be made within the retina of a single mouse.
Blinding	Investigators were blind to cell identity during morphological analysis (confocal IHC and EM reconstruction). Investigators were not blind to analysis of physiological responses as the cell types were identified by their unique physiology and morphology per previous publications.

Reporting for specific materials, systems and methods

We require information from authors about some types of materials, experimental systems and methods used in many studies. Here, indicate whether each material, system or method listed is relevant to your study. If you are not sure if a list item applies to your research, read the appropriate section before selecting a response.

Materials & experimental systems

n/a	Involved in the study
☐	☒ Antibodies
☒	☐ Eukaryotic cell lines
☒	☐ Palaeontology and archaeology
☐	☒ Animals and other organisms
☒	☐ Clinical data
☒	☐ Dual use research of concern
☒	☐ Plants

Methods

n/a	Involved in the study
☒	☐ ChIP-seq
☒	☐ Flow cytometry
☒	☐ MRI-based neuroimaging

Antibodies

Antibodies used	Rabbit anti-PSD95, Cell Signaling #3450<br>Donkey anti-rabbit Alexa Fluor 647, Jackson Immuno #711-605-152<br>Goat anti-ChAT, Millipore #AB144P<br>Donkey anti-goat Alexa Fluor 647, Life Technologies #A21447<br>Mouse anti-SMI-32, BioLegend #801702<br>Donkey anti-mouse Alexa Fluor 647, Life Technologies #A31571<br>Streptavidin 488, Thermo Science #21832
Validation	Rabbit anti-PSD95 (Cao, J. et al. (2005) J. Cell Biol 168, 117-26.; Chetkovich, D.M. et al. (2002) J. Neurosci. 22; 6415-25., Cai, C. et al. (2006) J. Biol. Chem. 281, 4267-73.; Yao, W.D. et al. (2004) Neuron 41, 625-38.; Cline, H. (2005) Curr. Biol. 15, R203-5.)<br>Goat anti-ChAT (Jin, M. et al. (2015) Molecular brain; Johnson, V et al. (2015) PloS one; Tikidji-Hamburyan, A., et al. (2015) Nature neuroscience; Li, S., et al. (2015) The Journal of neuroscience; Whitney, IE., et al. (2014) The Journal of neuroscience)<br>Mouse anti-SMI-32; Tan H, et al. (2022) J Comp Neurol. doi:10.1002/cne.25275; Gao J., et al. (2022) J Comp Neurol. doi:10.1002/cne.25293; Kowal TJ., et al. (2022) J Comp Neurol. doi:10.1002/cne.25326)

Animals and other research organisms

Policy information about studies involving animals; ARRIVE guidelines recommended for reporting animal research, and Sex and Gender in Research

Laboratory animals	Mice aged 6 - 36 weeks were used. For experiments requiring labeled type 6 BCs (Figs. 4-5 and Supplementary Fig. 7), CCK-ires-Cre/Ai14 mice were used (Jackson Lab Strain # 012706 / 007914). All other experiments used wild-type mice (C57BL/6, Jackson Lab Strain # 000664).<br>Mice were housed in a 14:10 dark light cycle (14 hours of darkness followed by 10 hours of light). Experiments were performed during dark hours. Housing temperature ranged from 21 °C to 23 °C. Housing humidity ranged from 30% to 70%.
Wild animals	No wild animals were used.
Reporting on sex	Mice of either sex were used with random selection. Sex was not recorded.
Field-collected samples	No field samples were collected.
Ethics oversight	Animals were used and cared for in accordance with protocols approved by Northwestern University Institutional Animal Care and Use Committee

Note that full information on the approval of the study protocol must also be provided in the manuscript.

Plants

Seed stocks	No plants were used.
Novel plant genotypes	No plants were used.
Authentication	No plants were used.
